# Effect of Superabsorbent Polymer (SAP) Size on Microstructure and Compressive Strength of Concrete

**DOI:** 10.3390/polym16020197

**Published:** 2024-01-09

**Authors:** Xiaobo Niu, Yile Zhang, Yogarajah Elakneswaran, Miyu Sasaki, Takeshi Takayama, Hajime Kawai

**Affiliations:** 1 Division of Sustainable Resources Engineering, Faculty of Engineering, Hokkaido University, Sapporo 060-8628, Japan; 2 Industrial & Household Chemicals Research Department, Industrial & Household Solutions Division, Nippon Shokubai, Osaka 564-0034, Japan; miyu_sasaki@shokubai.co.jp (M.S.);

**Keywords:** superabsorbent polymer, hydration rate, porosity, compressive strength, concrete, size

## Abstract

Superabsorbent polymers (SAPs) are hydrophilic, polymeric network materials renowned for their ability to enhance various properties of cementitious materials. This investigation examines the impact of SAP size on the hydration degree, porosity, and compressive strength of cement pastes and concrete under diverse curing conditions and ageing periods. The findings reveal that SAP addition stimulates the hydration of the C_2_S phase, particularly during the early curing stages, thereby favouring early strength development. However, the effect of SAPs on hydration promotion diminishes as their size increases. Conversely, the size of SAPs affects the hydration range of their action, and the 400 µm SAP demonstrates the most extensive range of hydration enhancement, reaching up to 105 µm. Additionally, SAPs effectively reduce porosity in small pores (4 nm–10 μm), with 200 μm and 400 μm SAPs exhibiting the highest efficacy. While analysing the effects of SAPs on larger pores (>10 μm), the results show that although larger SAPs result in larger average porosity, the total porosity is effectively reduced, particularly in samples incorporating 400 μm SAP. The compressive strength of cement paste, even after 28 days, is slightly reduced following the introduction of SAPs. However, the strength of concrete, due to the naturally occurring pores eliminating the negative effects of the pores produced by SAPs, is significantly increased following the introduction of SAPs, especially 400 µm SAP.

## 1. Introduction

Superabsorbent polymers (SAPs) are highly hydrophilic polymeric network materials that exhibit significant potential in absorbing and storing large amounts of water or aqueous solutions, and they have found wide applications in numerous fields [1]. In recent years, the research and application of SAPs as internal curing agents in cementitious materials have received considerable attention, especially in low water-to-cementitious-materials ratios such as Ultra-High Performance Concrete (UHPC) and High-Performance Concrete (HPC) [2,3,4]. The water-holding capacity of SAPs allows them to provide a continuous internal water source for the hydration reactions of cement and serve as a supplementary water source during the concrete curing process under low relative humidity conditions when water is insufficient due to evaporation [5]. Previous studies have shown that the addition of SAPs during curing can effectively improve the autogenous shrinkage of concrete [6,7,8], enhance freeze–thaw resistance [9], reduce drying shrinkage [10,11], increase self-healing capacity, minimize the formation of microcracks [12], and enhance the durability of cementitious materials [6].

The impact of adding SAPs on the compressive strength of cementitious materials has been a subject of controversy in previous studies. Some research has suggested that the inclusion of SAPs could lead to a reduction in the compressive strength of concrete [12,13]. This viewpoint is primarily based on the notion that additional water needs to be added to maintain the plasticity of concrete, which consequently increases the pore water content and weakens its strength [14,15,16,17]. Simultaneously, the introduction of SAPs creates additional voids, further compromising the strength of cement [18,19]. However, reports have also suggested that adding SAPs can enhance the strength of cementitious materials [13,17]. The positive effect is attributed to SAPs acting as internal curing agents, continuously providing a water source that promotes hydration. Consequently, this counteracts the negative impact caused by the internal voids created by SAPs [20].

Generally, the influence of SAPs on the strength of cementitious materials depends on several factors, such as the curing period, curing environment, and properties of SAPs. Previous studies have indicated that SAP-added cement mortars cured for 28 days exhibit higher compressive strength than those with shorter initial curing periods of 3 or 7 days [21]. Several other studies have found significant changes in self-shrinkage due to changes in internal relative humidity during conservation, resulting in variations in hydration outcomes and compressive strength [22,23,24,25,26]. Variations in the use of SAPs can also cause significant changes in the properties of cementitious materials. Different dosages can result in varying water-to-cement ratios and pore structures, thereby affecting the strength differentially [18]. On a dosing basis, whether the SAPs used are pre-saturated or not will also have a direct impact on the plasticity as well as the strength of the cement [27,28,29,30]. In our previous study, a delayed absorption type of SAPs (I-SAP) was proposed with only minimal changes in rheology while significantly improving the strength of concrete and cement paste [20]. However, previous studies have not yet investigated the impact of the size of I-SAP on the internal curing capacity of cementitious materials. It is worth noting that size is one other key factor, as smaller SAPs can more effectively occupy the pore space within the concrete, potentially leading to different strength effects [13,16]. SAPs of the same type but varying in size exhibit distinct water release capabilities. The larger the size, the wider the range of influence, resulting in higher hydration rates and a larger area of self-healing space [31,32]. Additionally, it is important to consider that the volume of the SAP itself generates pores, which can potentially reduce the overall strength of the material [33,34].

In order to gain further insights and enhance the efficacy of delayed absorption I-SAP in the internal curing of cementitious materials, this study investigates the impact of different I-SAP sizes (ranging from 80 μm to 1 mm) made of cross-linked modified acrylate. The investigation is conducted under varying environmental conditions, including water, sealed, and RH60% environments. The evaluation of different I-SAP sizes on internal curing involved analysing the hydration degree, porosity, and microstructure of cement paste while also comparing the compressive strength of the cement paste with that of concrete. The findings of this research contribute to an enhanced understanding of the size factor of SAPs and bear significant implications for the internal curing of cementitious materials. 

## 2. Materials and Methods

### 2.1. Materials and Sample Preparation

Cement paste and concrete mixed with superabsorbent polymers (SAPs) were prepared using ordinary Portland cement (OPC) from Taiheiyo Cement, Tokyo, Japan. The chemical composition of the OPC is provided in Table 1. This study used a delayed absorption I-SAP composed of cross-linked modified acrylates (Nippon Shokukai, Osaka, Japan). SAP particles with average sizes (d50) of 80, 200, 400, and 1000 μm were chosen to investigate the influence of SAP particle size on internal curing efficiency. Detailed information about the samples is found in Table 2. SAP was added at a rate of 0.3% by weight of cement. Moreover, cement paste was prepared with a water-to-cement ratio of 0.45. A high-performance air-entrained (AE) water-reducing agent and deformer (DEF) were added to achieve the desired water-to-cement ratio and flow value. The cement and water were mixed in a mortar mixer for 60 s at a low speed, followed by 120 s at a high speed. The resulting cement paste was stored in a plastic bottle and periodically agitated with a spatula every hour for 7 h to prevent bleeding. Afterwards, the cement paste was poured into moulds measuring 50 mm in diameter and 100 mm in height. The moulds were sealed using a plastic sheet and stored at a constant temperature of 20 °C for 24 h. The samples were removed from the moulds and divided into three groups for further curing: water immersion, sealed conditions, and a relative humidity (RH) of 60%. The specimens were cured at 20 °C for 3, 7, and 28 days. Subsequently, various measurements were performed on the cured samples. The mix proportions of concrete and its fresh mix characteristics refer to previous studies [20].

### 2.2. Experimental Procedure

#### 2.2.1. Evaluation of Hydration

The cement paste samples were shattered into pieces and placed in acetone for 24 h to halt the hydration reaction after undergoing a specific curing time [35,36]. After removing acetone through vacuum filtration, the samples were then stored in a 40 °C thermostat until their weights were stabilized, signifying the absence of acetone. To determine the extent of unhydrated cement necessary for calculating the rate of hydration reaction, a portion of the sample was ground into a powder with particle sizes below 150 μm for X-ray diffraction (XRD) measurements. The 10 wt% corundum (α-Al_2_O_3_) was used as an internal standard substance. XRD analysis was conducted using a Rigaku MultiFlex X-ray generator (Tokyo, Japan) with the following parameters: tube voltage of 40 kV, tube current of 40 mA, scanning speed of 6.5° min^−1^, scanning range of 2θ = 5–70°, step width of 0.02°, divergence slit of 0.5°, scattering slit of 0.5°, and reception slit of 0.3 mm. Previous studies have indicated that the impact of SAP on the hydration of belite (α-C_2_S) in cement hydration products is particularly significant due to its delayed internal hydration effect [20]. Therefore, belite was chosen as the representative hydration reaction product in this study.

Furthermore, after undergoing a process that included the cessation of hydration and the removal of acetone, a subset of the samples that had been cured for 28 days was cast with epoxy resin and subjected to a polishing procedure employing sandpaper of various grid sizes, including 80, 240, 480, 1000, and 1500. The specimens were further polished using diamond paste with a roughness of 0.25 μm following surface drying and platinum sputtering. The treated samples were examined using a Scanning Electron Microscope (SEM, JSM-IT200, JEOL, Tokyo, Japan) to assess the extent to which the hydration process was affected by SAP. Measurements were conducted in backscattered electron (BSE) image mode, operating at an accelerating voltage of 15 kV. The resulting BSE images were divided into two fractions: the fraction representing unhydrated cement and other components. This division was accomplished using brightness [37], and, subsequently, the images were binarized. The degree of unhydration was calculated using Equation (1).

(1) $$D\left(\%\right)=\frac{A}{255}\times 100(\%)$$

where *D* is the degree of unhydration (%), *A* is the average grey value of the unhydrated area, and 255 is the maximum grey value.

#### 2.2.2. Porosity Measurement

In cement paste specimens, there are two categories of porosities: microporosity, which is smaller than 10 μm and results from uneven hydration reactions and shrinkage; and voids larger than 10 μm that are caused by the presence of SAP. To determine the distribution of micropores, a mercury intrusion pore size meter (MIP) was utilized. The hydrated samples were cut into small pieces measuring approximately 5 mm and subjected to vacuum drying. The MIP testing parameters included a surface tension of 485 erg/cm^2^, a contact angle of 130°, and a pressure of up to 4.45 psi, enabling the measurement of pores with diameters larger than 3 nm. To assess the presence of air voids, a linear transverse method was employed on hardened specimens of each mixture in accordance with ASTM C457/C457M [38].

In contrast, the voids attributed to SAP were observed using microfocus X-ray computed tomography (µCT) on cylindrical cement paste specimens. Because of the need to detect cavities caused by SAP, these 28-day long-cured specimens were selected for examination. The specimens were 50 mm in diameter × 40 mm in height in size. The void structures were scanned using the “TOSCANER 31300µhd” µCT scanner, manufactured by Toshiba Information Technology & Control Systems Co., Ltd. in Tokyo, Japan. Cone beam mode was employed for the scans, with a tube voltage of 130 kV and a tube current of 124 µA as the scanning parameters. In all scans, 1500 projection directions were utilized. In each projection direction, 20 consecutive scans were conducted, and the averaged projection data were used for image reconstruction to reduce the statistical noise caused by the X-ray image intensifier. The resulting μCT images had dimensions of 1024 × 1024 voxels, with each voxel measuring 51 μm × 51 μm × 77 μm [39]. During the analysis, the virtual specimen was generated from 16-bit cross-sectional images acquired from μCT. The pixel value within the range of 0–65,535 (2^16^-1) represents the relative density of the phase present in the specimens.

#### 2.2.3. Compressive Strength Measurement

The compressive strength of the hardened cement paste and concrete, which underwent a 28-day curing process, was measured following JIS A 1108 for cement paste and JIS A 1132 for concrete, respectively. To conduct the measurements, a cylindrical specimen with dimensions Φ50 mm × 40 mm was utilized, as specified by the instrument. The calculation for determining the pressure is described by Equation (2):

(2) $$f_{c}=\frac{P}{\pi \times (\frac{d}{2}{)}^{2}}$$

where *f_c_* is compressive strength (N/mm^2^), *P* is maximum load (N), and *d* is sample diameter (mm).

## 3. Results and Discussions

### 3.1. Hydration

**Figure 1:**
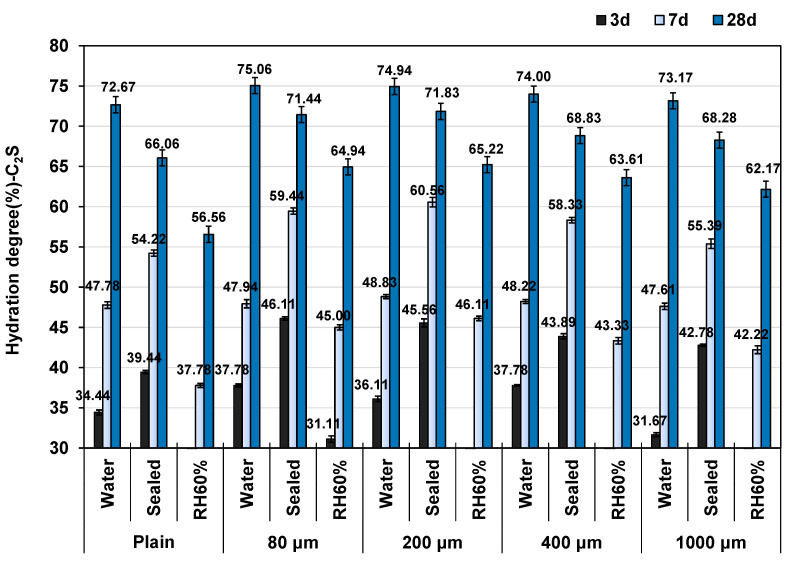
Effect of SAP sizes and curing conditions on C_2_S hydration with different curing period.

Figure 1 illustrates the influence of various sizes of SAP on the hydration degree of cement paste under different conditions and to varying curing durations. The hydration degree is indicated by the results of the belite by XRD/Rietveld analysis, as previous studies have suggested that the belite phase plays a crucial role in the impact of SAP on cement hydration. This phase, known for its slow reaction rate compared to other phases, is particularly susceptible to the internal delayed influence curing of SAP [20,40,41]. The results show that the hydration degree of C_2_S varies depending on different curing conditions. Following a 3-day curing period, the group under sealed conditions demonstrated the highest hydration degree. The specimens cured in water showed an inferior hydration degree, whereas those kept in an RH 60% environment showed almost no development of hydration. Similarly, after 7 days of curing, the order of hydration degree remained consistent, with the sealed group exhibiting the highest hydration, followed by the water group. However, even though they still indicated the lowest hydration degree of all environments, specimens cured at RH 60% showed some hydration development at this stage. After a 28-day curing period, specimens kept in water exhibited the highest hydration degree, while specimens kept at RH 60% continued to show the lowest hydration degree.

After the introduction of SAP, the relative relationship between the hydration degree of C_2_S in various curing environments remained unchanged. Nevertheless, adding SAP to the specimens led to a higher degree of C_2_S hydration in the cement paste across all curing conditions compared to the control group. Additionally, the extent of hydration increase gradually diminished as the size of SAP addition increased. Remarkably, SAP exhibited a significant promotion of C_2_S hydration in the samples after a 3-day curing period, consequently enhancing the early strength of the cement material. 

**Figure 2:**
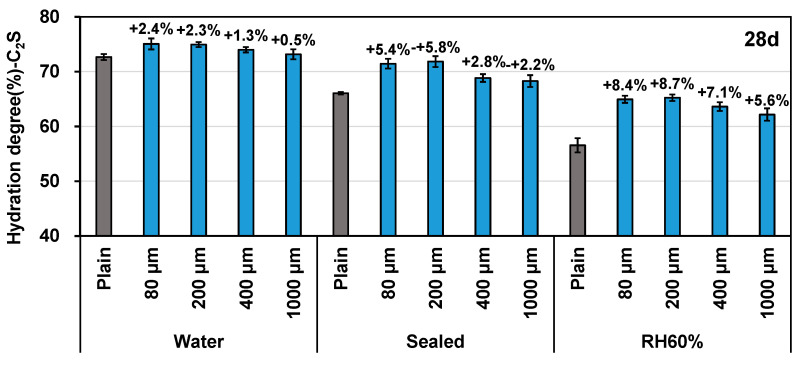
Effect of SAP sizes and curing conditions on C_2_S hydration at 28 days.

When focusing on the results of 28 days of curing (Figure 2), it is evident that the hydration degree results follow the order of the water group, the sealed group, and the RH60% group, with or without the addition of SAP. However, the improvement in the hydration results by adding SAP was in the opposite order, i.e., the addition of SAP promoted the hydration of C_2_S of the cement samples in the RH60% condition most significantly. In an identical curing environment, no notable distinction in the impact on hydration outcomes was observed between 80 μm and 200 μm SAP. However, as the size of the SAP increased incrementally, its efficacy in promoting hydration significantly declined.

**Figure 3:**
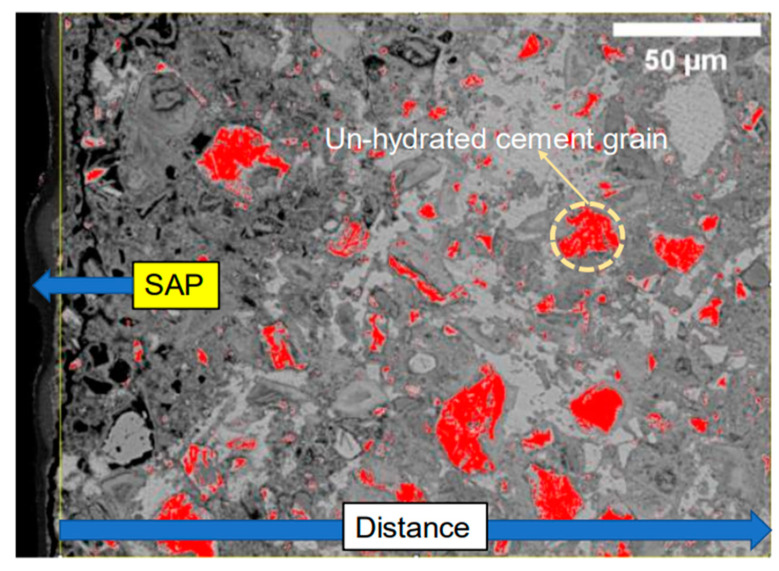
Schematic diagram of SEM-BSE image processed by ImageJ. (Unhydrated cements are depicted in red, while the grey areas signify hydration products. The black areas delineate microporosity within the structure).

**Figure 4:**
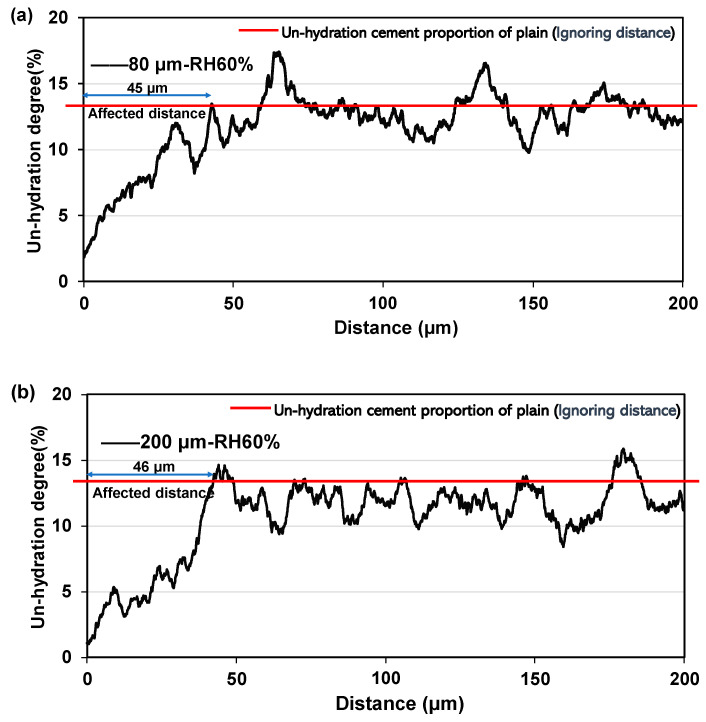

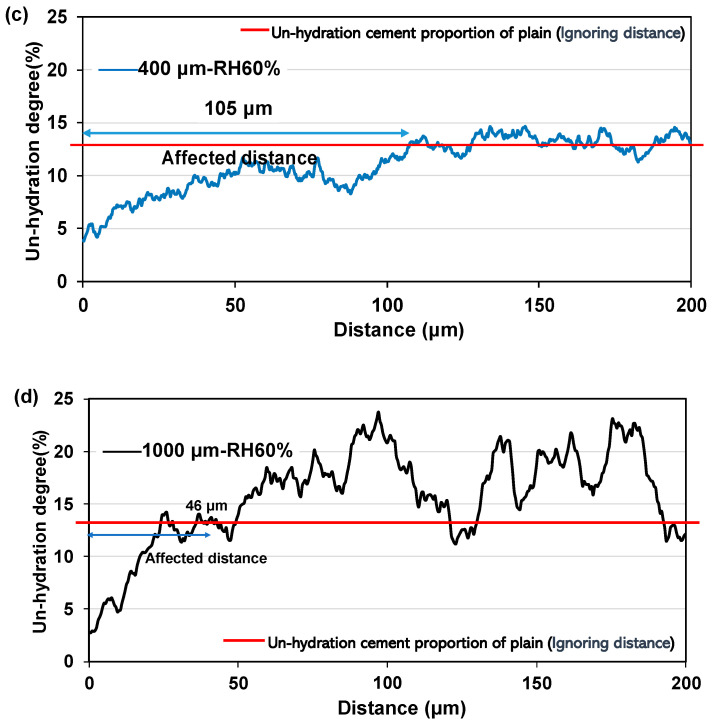
Range of hydration proportions influenced by SAP of (**a**) 80, (**b**) 200, (**c**) 400, and (**d**) 1000 μm. (The blue arrows indicate range of average hydration effects).

Backscattered electron (BSE) images were utilized to analyse the influence of different sizes of SAP on the hydration of cement paste specimens after 28 days of curing under an RH60% environment. The images processed through ImageJ software (version 1.54a) are displayed in Figure 3, where unhydrated cements are identified based on their grayscale values and highlighted in red. The left side of the figure shows the SAP marked by a black border, while other black areas represent microporosity. The variation in the fraction of unhydrated cement from the SAP boundary is depicted in Figure 4. As the distance from the SAP surface increased, the proportion of unhydrated cement gradually rose until it reached a plateau, matching the average proportion observed in the control group without SAP addition. Notably, the influence and promotion of hydration showed no significant disparity among SAP sizes of 80, 200, and 1000 μm, as the proportion of unhydrated cement levelled off at distances of 45, 46, and 46 μm from the SAP surface, respectively (Figure 4a,b,d). Conversely, the 400 μm SAP demonstrated a considerable range of influence, promoting hydration up to 105 μm (Figure 4c). It is worth emphasizing that although the unhydration of the samples with 1000 μm SAP exhibited a tendency towards a plateau, there was an overall increase when compared to the control group. This can be attributed to potential measurement inaccuracies stemming from the formation of substantial voids resulting from the considerable shrinkage of the larger-sized SAP during the drainage process. These voids, being of a considerable size, may affect the accuracy of grayscale recognition, thus contributing to the observed increase in unhydration.

### 3.2. Porosity

Two distinct types of pores were observed in the cement paste specimens with SAP addition. The first type comprises inherent pores that form during the hydration process, influenced by the characteristics of the cement. These pores are typically small, ranging in diameter from 4 nm to 10 μm [42]. Their formation is primarily governed by factors such as the degree of hydration and shrinkage. Conversely, the second type of pores is relatively larger and arises from the shrinkage of SAP following water discharge. These pores generally exceed 10 μm in diameter [20].

**Figure 5:**
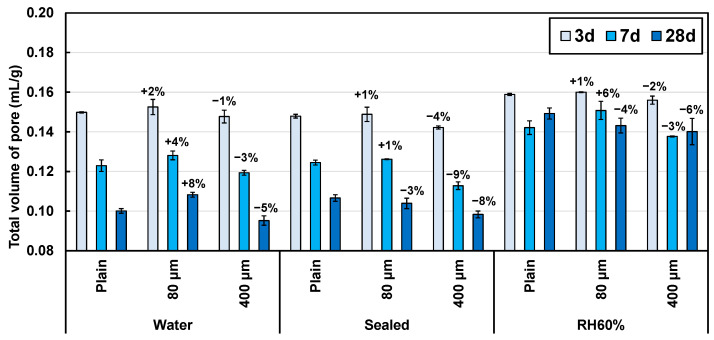
Total volume of small pores (4 nm–10 μm) in cement paste after curing for 3, 7, and 28 days under different curing conditions.

The porosity of small pores in the range of 4 nm–10 μm obtained by MIP measurements is shown in Figure 5. Samples cured in water and sealed condition exhibited close porosity and gradually decreased with increasing curing time, consistent with the hydration results (Figure 1). It has also been previously demonstrated that the porosity of cementitious materials decreases with increasing curing humidity [43]. However, what seems to contradict the hydration results is that the porosity of samples with 400 μm SAP cured under these two conditions is obviously reduced compared to those with 80 μm SAP, which indicates that the 400 μm SAP effectively inhibits the shrinkage of the cement and thus reduces the porosity. The 28-day curing porosity of the cement paste specimens maintained at RH 60% was higher than 7d, which could be attributed to the significant shrinkage produced by the cement paste cured in this environment. However, after adding SAP, the shrinkage was suppressed, thus reducing the porosity to varying degrees.

**Figure 6:**
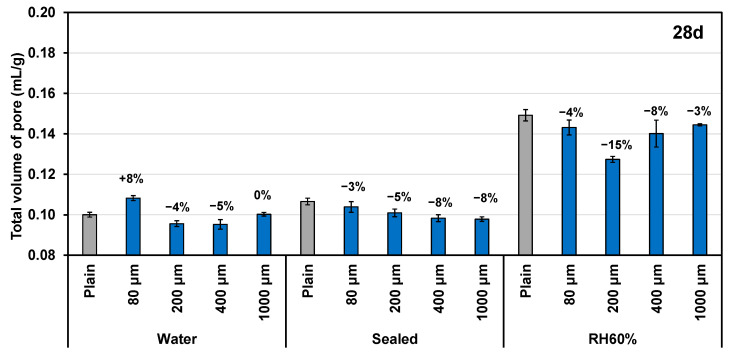
The total volume of small pores (4 nm–10 μm) in the cement paste after 28 days of curing under different curing conditions.

Figure 6 illustrates the porosity of small pores in the range of 4 nm–10 μm after 28 days of curing under various sizes of SAP admixture and different curing conditions. The incorporation of SAP proved effective in reducing the porosity of the cement paste samples in most cases. However, for the samples cured in water with the addition of 80 μm SAP, the measurements might have been inaccurate due to the inherently low porosity of the samples under such conditions. Nevertheless, this result clearly demonstrates the capability of SAP to reduce the porosity of cement paste following long-term (28 days) curing. Moreover, the effectiveness of SAP in reducing the porosity of cement paste also varies with the size of the SAP particles. SAPs with sizes of 200 μm and 400 μm displayed greater efficacy in decreasing the porosity of cement paste compared to SAPs of 80 μm and 1000 μm. The 200 μm SAP exhibited a slightly greater reduction in porosity in both water and an RH 60% conditioning environment, while the 400 μm SAP demonstrated more significant reduction under sealed conditions. These findings highlight the influence of the particle size of SAP on its ability to reduce porosity in cement paste.

**Figure 7:**
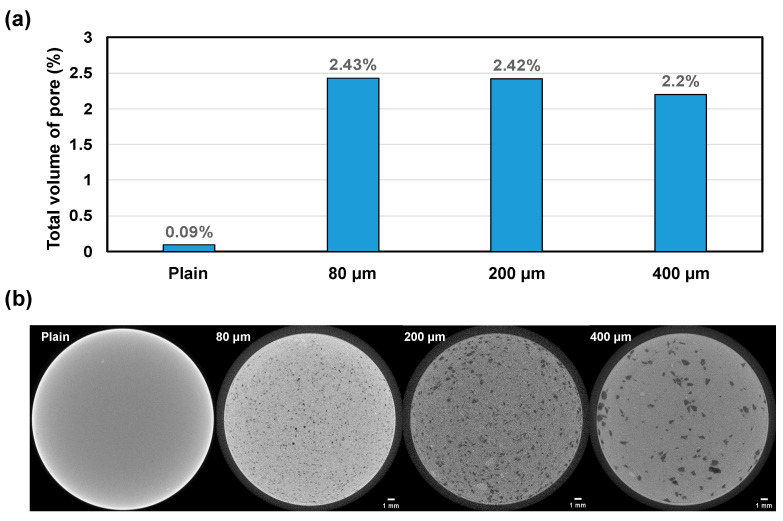
(**a**) The total volume of large pores (>10 μm) and (**b**) the images of μCT cross-section in the cement paste after 28 days of curing under RH60% conditions.

Figure 7 illustrates the presence of pores larger than 10 μm in diameter resulting from the volume shrinkage of SAPs, as determined by μCT. In the plain cement paste, only a negligible number of pores larger than 10 μm were detected, indicating that pores of this size generated by the hydration and shrinkage process of the cement paste are minimal (Figure 7a). These results are also confirmed by a number of experimental and empirical equations [44]. However, upon the addition of SAP, the presence of distinct large pores larger than 10 μm can be observed in the μCT cross-sections (Figure 7b). The average size of these large pores increases with the size of the added SAPs. Notably, it is important to highlight that the total volume of large pores decreases with the increasing size of SAPs. These findings suggest that although the 400 μm SAP induces larger average pore sizes compared to the 80 μm and 200 μm SAPs, it effectively reduces the overall volume of large pores resulting from the addition of SAPs. This phenomenon is attributed to the ability of the 400 μm SAP to influence the maximum range of cement hydration, thereby effectively impeding shrinkage initiated from the surface of SAPs and maximizing the inhibition of further expansion of large pores caused by the SAPs, as evidenced by the extent of hydration influence results (Figure 4).

### 3.3. Compressive Strength

**Figure 8:**
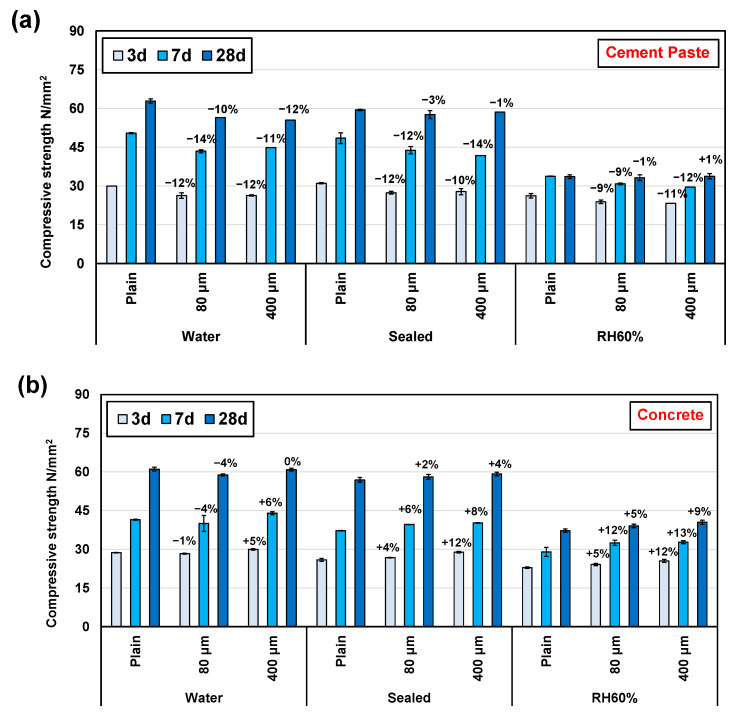
Compressive strength of (**a**) cement paste and (**b**) concrete under different curing conditions.

According to previous research [20], the addition of SAPs significantly impacts the flowability of cement mixtures, leading to a considerable decrease. To maintain a relatively stable rheology, additional water is required. However, this increases the water-to-cement ratio, which is one of the factors contributing to the reduction in the expected strength of hardened cement or concrete. Additionally, the release of water from SAPs creates large pores that greatly diminish the compression strength of the cement (Figure 7). Figure 8a demonstrates that the inclusion of SAPs in the cement paste reduced its compression strength. And the effect is not evident between 80 μm and 400 μm of SAPs. A decrease of approximately 10% was evident after 3 days of curing. However, it is astonishing to discover that as the curing period extended, the decrease in compression strength after 28 days of curing became less pronounced, especially for the paste cured at 60%RH. In fact, the 28-day-cured paste at 60%RH exhibited a slight increase in strength for the sample with 400 μm SAPs added. This can be attributed to the internal curing conditions offered by the release of internal water from the SAPs. These conditions contribute to enhanced cement hydration, the inhibition of shrinkage, and reduction in the formation of small pores (Figure 6). Such factors favourably influence the compressive strength of the cement paste, effectively counteracting the negative effects of SAP addition [10]. Furthermore, after accounting for these offsetting factors, the final compressive strength varies depending on the curing conditions. Samples cured in water showed no significant change in the reduction of compression strength due to the ample availability of water, with minimal positive effects from internal water curing being provided by SAPs. On the other hand, the reduction in compression strength for samples aged 28 days in a sealed environment was notably mitigated due to reduced water availability. This effect was even more pronounced in samples cured at 60%RH. However, the effect of 80 μm and 400 μm SAPs on the final compressive strength of the cement paste was not significant.

Figure 8b presents the results of the compressive strength analysis of concrete after adding SAPs. In contrast to cement paste, the incorporation of SAPs resulted in a marked escalation in the compressive strength of concrete. This enhancement became increasingly prominent as the moisture content within the curing environment decreased, while an inverse relationship was observed concerning the curing period. This phenomenon can be ascribed to the complex composition of concrete, involving a blend of diverse raw materials, including aggregates of varying grades. Consequently, inherent voids exist within the concrete, particularly during its initial hydration stage. These inherent voids possess a larger average size and total porosity compared to the voids introduced by SAPs during the early stages of hydration when the concrete structure has not yet achieved a relatively dense formation. Thus, the detrimental impact on concrete’s compressive strength due to SAP addition is relatively negligible in comparison to its effect on cement paste. Conversely, the overall compressive strength of the concrete is fortified owing to heightened internal hydration; nevertheless, this enhancement experiences a slight decline with an increase in the hydration period. Notably, 400 μm SAPs exhibit a more pronounced effectiveness in augmenting the compressive strength of concrete in comparison to 80μm SAPs, aligning with both the hydration range (Figure 4) and porosity results (Figure 5).

Table 3 outlines the varied impacts of distinct particle sizes of SAPs on cementitious materials. A comprehensive overview of these details can be found in the Conclusion section.

## 4. Conclusions

This study aims to explore and analyse the factor of size that influences the internal curing effects of I-SAP in cementitious materials. This investigation involved evaluating the degree of hydration, porosity, and microstructure of cement pastes at various curing environments and ages. Additionally, a comparison was made between the compressive strength of cement pastes and concrete. The specific findings are as follows:

➢ Incorporating SAPs into cement paste results in heightened hydration levels within the C_2_S phase, thereby increasing the initial strength. The size of SAP particles plays a pivotal role in determining the degree of hydration augmentation, with larger SAP particles demonstrating a diminishing impact on enhancing hydration. Among all sizes, the 400 µm SAP showcases the most extensive scope of promoting hydration influence at the 105 µm range.
➢ The introduction of SAPs effectively diminishes the porosity within small pores (4 nm–10 μm) in cement paste. SAPs with sizes of 200 μm and 400 μm exhibit superior effectiveness compared to 80 μm and 1000 μm SAPs. However, the inclusion of SAPs can instigate the creation of larger pores (>10 μm) due to the release of water and subsequent shrinkage of the SAPs themselves. While the average size of these larger pores correlates with the larger sizes of SAPs, the overall porosity of these samples with larger SAPs gradually decreases, notably in samples containing 400 μm SAPs. This occurrence can be attributed to the range of promotion hydration effects exerted by SAPs.
➢ The addition of SAPs into cement paste initially causes a notable decrease in compressive strength due to the introduction of release-shrinkage pores. However, this effect lessens over time, since the internal curing conditions provided by SAPs improve hydration, curb shrinkage, and reduce small pore formation. The compressive strength of SAP-introduced cement paste notably varied based on curing moisture levels. Samples cured in RH60%, with lower moisture content, exhibited a positive effect. In contrast, adding SAPs to concrete enhanced compressive strength, particularly in shorter curing periods and drier environments. Among the various SAP sizes, the 400 µm SAPs significantly bolstered the compressive strength of concrete samples, most notably in the early hydration stages.

## Figures and Tables

**Table 1:** Chemical composition of OPC.

Chemical Composition	Mass (%)
CaO	67.07
SiO_2_	20.38
Al_2_O_3_	5.12
Fe_2_O_3_	3.05
SO_3_	1.86
MgO	1.30
K_2_O	0.36
Na_2_O	0.30
TiO_2_	0.29
P_2_O_5_	0.20
MnO	0.05

**Table 2:** Sample preparation and curing condition.

W/C	SAP Addition (%/Cement)	SAP Size (µm)	AE (%/Cement)	DEF (%/Cement)	Curing Condition
0.45	-	-	0.012	7.7	Water
					Sealed
					RH60%
	0.3	80	0.022		Water
					Sealed
					RH60%
		200			Water
					Sealed
					RH60%
		400			Water
					Sealed
					RH60%
		1000			Water
					Sealed
					RH60%

**Table 3:** Comparison of SAP particle size for each property.

	80 μm	200 μm	400 μm	1000 μm
Degree of hydration (28 d)	Optimal and similar promotion		Slightly reduced promotion	Reduced promotion
Hydration range (28 d)	Around 45 μm	Around 45 μm	105 μm	Around 45 μm
Small porosity	−4%~+8%	−15%~−4%		−3%~0%
Large porosity	+2.43%	+2.42%	+2.2%	N/A
Compressive strength	−10%~−1% in cement −4%~+5% in concrete	-	−12%~+1% in cement 0%~+9% in concrete	-

## Data Availability

Data are contained within the article.
